# Publisher Correction: Using smartphones to optimise and scale-up the assessment of model-based planning

**DOI:** 10.1038/s44271-023-00047-4

**Published:** 2023-12-16

**Authors:** Kelly R. Donegan, Vanessa M. Brown, Rebecca B. Price, Eoghan Gallagher, Andrew Pringle, Anna K. Hanlon, Claire M. Gillan

**Affiliations:** 1 School of Psychology, Trinity College Dublin, Dublin, Ireland; 2 Trinity College Institute of Neuroscience, Trinity College Dublin, Dublin, Ireland; 3 Department of Psychiatry, University of Pittsburgh School of Medicine, Pittsburgh, USA; 4 Global Brain Health Institute, Trinity College Dublin, Dublin, Ireland

**Keywords:** Human behaviour, Research data

Correction to: *Communications Psychology* 10.1038/s44271-023-00031-y, published online 01 November 2023.

The original version of this Article contained an error in Fig. 3, panel e, in which the asterisks that signify significance for the conditions “Eating Disorder” and “Impulsivity” were replaced by exclamation marks. The original version of the Article also contained an error in Fig 5, panel c.ii in which the x-axis and the label “women” were shifted upward and out of line with the rest of the axis and labels where the binned trials that show the model-based association with gender are displayed. The original article has been corrected.

