# Supplementary material for: Using smartphones to optimise and scale-up the assessment of model-based planning
Source: Commun Psychol. 2023 Nov 1;1:31. doi: 10.1038/s44271-023-00031-y (PMC11332031; doi:10.1038/s44271-023-00031-y)
Supplement: Supplementary file 1 — Supplementary Materials [file 44271_2023_31_MOESM1_ESM.pdf]

Supplementary Materials

Supplementary Methods 3
Supplementary Method 1. Model-Free Estimates, Reliability and Relationship to Model-Based Planning 3
Supplementary Method 2. Assessing the impact of an error in STAI-T administration 3
Supplementary Method 3. Computational Modelling 3
Supplementary Method 4. Comparing different estimation approaches 6
Supplementary Notes 8
Supplementary Note 1. Information Sheet for The Neureka project In-App 8
Supplementary Figures/Tables 11
Figure S1. Passive instructions from Cannon Blast shown to participants. 11
Figure S2. Two-step reinforcement learning task for assessing model-based learning. 12
Figure S3. Reward trajectories for each of the ten drifting reward probabilities sets 13
Figure S4. Within-task correlations in our dataset and other publicly available datasets 14
Supplementary Tables 15
Table S1. Task Parameters used in Traditional Two-step Task and Cannon Blast 15
Table S2. Descriptive Information of Easy and Medium Levels of Difficulty in Cannon Blast 16
Table S3. Demographic Characteristics in Experiment 2 in the Whole Sample and the two Age, Gender and Education Matched Samples 17
Table S4. Mental Health Summary in Experiment 2 (N=1451): Mean symptoms and their association with age, gender, and education. 18
Table S5. Descriptive Information of those with Repeated Plays of Cannon Blast 19
Table S6. Descriptive Information of Reward Drift Sets assigned at Each Block 20
Table S7. Logistic Regression Analysis across various Task Modifications 21
Table S8. Associations between Model-based Planning and Individual Differences (N=5005) and Clinical Associations (N=1451) 22
Table S9. Associations with Model-free, Model-based, Stay and Transition estimates from Cannon Blast with Individual Differences and Clinical Associations 23
Table S10. Linear Regressions Predicting Stay Behaviour by Reward, Transition and Diamond Hit on the Previous Trial. 24
Table S11. Linear Regressions assessing the External Validity across various Task Modifications 25
Table S12. Full Comparison Models across Task Modifications 26
Table S13. Intraclass Correlation Co-efficient (ICC) testing the Test-retest Reliability of Model-based Indices across Task Difficulty/Order 27
Table S14. Assessing the Impact of Increasing Trial Number per Participant on Mean-level Model-based Indices and their Reliability (N=716) 28

Table S15. Logistic Regression Analysis with Increasing Trial Number (N=716) 29
Table S16. Linear Regressions assessing the External Validity with Increasing Trial Number (N=716) 31
Table S17. Full Comparison Models with Increasing Trial Number (N=716) 33
Table S18. Candidate Model Comparison and Evaluation for Hierarchical Bayesian Models 34
Table S19. Comparing the Association between Model-based Index and Individual Differences using Different Analytic Approaches to Calculate Model-based Index .35
Supplementary References 36

Supplementary Methods

Supplementary Method 1. Model-Free Estimates, Reliability and Relationship to Model-Based Planning

MFI was less consistent across tasks, with estimates from the Traditional task and Cannon Blast correlating at just r=.15. In the same sample, MFI from Cannon Blast correlated with both MFI and MBI estimates from the Traditional task, at comparable strengths (MFI-CB and MFI-Trad r=.15 & MFI-CB and MBI-Trad r=.19). This could suggest that the MFI estimate from Cannon Blast partially captures model-based processes. However, we urge caution in over-interpreting this result. The sample size for Experiment 1 was small (N=57) and we in fact observed larger correlations between model-based and model-free planning in the traditional task (r=.6) than in the gamified one (r=.06) in Experiment 1 (**Figure S3)**. In the larger citizen scientist dataset, the latter correlation rose to .33. Comparing these results to publicly available datasets, there is a relatively large range of values. For example correlation between MBI and MFI was .18 in Gillan et al.,[1] and .36 in Seow et al.,[2].

Supplementary Method 2. Assessing the impact of an error in STAI-T administration

A typographical error of the STAI questionnaire occurred affecting the users included in our analysis of clinical associations. The response options coded in the app reflected the STAI-State portion of the questionnaire rather than STAI-trait portion (State responses: 'Not at all', 'Somewhat', 'Moderately so', 'Very much so' as opposed to Trait responses: 'Almost never', 'Sometimes', 'Often', 'Almost always' respectively).

To check if this changed the meaning of the responses unduly, we compared the depression and STAI scores in our sample of N=1451 and compared it to previously found associations using the correct version. Prior research found the associations between SDS depression scores and STAI scores was r=.84 in 495 individuals[3]. In line with this, we found the association between SDS depression scores and STAI scores was r=.88. Additionally, as reported in the main text, factors derived from our dataset including participants with the incorrect version were all correlated with factors drawn from a dataset using the correct version in excess of .9, providing comfort that this error had no substantial impact of findings.

Supplementary Method 3. Computational Modelling

The basic reinforcement-learning (RL) models used in this study were based on those developed and refined across a range of studies modelling behaviour in the classic version of this task. We considered the 3 models tested previously considered in the context of validity of model-based assessments[4]. These were adapted to the fact that our task does not include a second stage decision as per[5]. For all models, Q values refer to the expected value of a given state. The first states are the two containers that can be selected, $a_{i,t}$*ai,t*, where *i* refers to the "mostly pink" or "mostly purple" containers and *t* refers to trial. The second states correspond to the colour of the ball fired by the cannon, pink or purple, denoted $s_{j,t}$*sj,t*, where *j* refers to pink or purple. Reward on a given trial is defined as the 'ball quality', i.e. whether or not it explodes ('good' or 'dud') prior to hitting its target, denoted r_t.

Model A: This model is based on the original paper describing this task[6] and consists of 5 free parameters, ω (the relative contribution of mode-based and model-free Q values on choice), $\alpha 1$ (the stage 1 learning rate), $\alpha 2$ (the stage 2 learning rate), ρ (the perseveration or 'stickiness' parameter) and β (the inverse temperature).

The model-free algorithm updates the Q values, $Q_{MF1}(a_{i,t})$, for two choices of containers ($a_{i,t}$) at stage 1 as follows: $Q_{MF1}(a_{i,t}) = Q_{MF1}(a_{i,t}) + \alpha 1 * (\delta_{1,t-1} + \delta_{2,t-1})$, where $\delta_{1,t}$ is the reward prediction error (RPE) associated with the chosen container ('first stage' in classic task): $Q_{MF2}(s_{j,t}) - Q_{MF1}(a_{i,t})$ and $\delta_{2,t}$ is the RPE associated with the ball (pink/purple) that was released ('second stage' in the classic task): $r_t - Q_{MF2}(s_{j,t})$. These Q values for the two ball colours are updated according to $Q_{MF}(s_{j,t}) = Q_{MF}(s_{j,t}) + \alpha 2 * (\delta_{2,t})$. The model-based algorithm applies a transition function, using known probabilities for how likely it is that a given ball is produced following a given container choice to ensure that terminal rewards are correctly assigned to initial choices. We assumed full knowledge of transitions in this task (80/20), as the proportion of pink/purple balls for each side was explicitly stated to subjects during the instructions and was visible throughout the experiment as the balls jumped around the two s. The model-based Q values for each container, $Q_{MB1}(a_{i,t})$, were thus computed as $Q_{MB}(a_{i,t})$ = $Q_{MF2}(s_{j,t}) * P(s_{j,t}|a_{i,t})$.To connect the values to choice of container, we use a Softmax choice rule, which assigns a probability to each action according to the weighted combination of the MB and MF estimates $Q_{net}(a_{i,t}) = \omega * Q_{MB}(a_{i,t}) + (1-\omega) * Q_{MF1}(a_{i,t})$. The probability of choosing each of the two containers is calculated, accordingly, as

$$P(a_{i,t}) = \frac{\exp\,[\beta *\ Q_{net}(a_{i,t}) + \rho * rep(a_{i,t})]}{\Sigma_{a'}(\exp\,[\beta * (\ Q_{net}(a'_t) + \rho * (rep(a'_t)])}$$

Model B: This model is based on that described in Otto, et al. [7] and consists of 4 free parameters, α (a single learning rate that is applied to both stages), ρ (the perseveration or 'stickiness' parameter) and βMF (the model-free inverse temperature) and βMB (the model-based inverse temperature). The model has two additional features compared to Model A: (i) rescaling of rewards (based on α), which is designed to reduce parameter collinearity and (ii) a decay or 'forgetting' of unselected parameters (based on 1- α). The former manifests in the RPE terms where $\delta_{1,t}$ i=$\frac{Q_{MF2}(s_{j,t})}{\alpha} - Q_{MF1}(a_{i,t})$ and $\delta_{2,t}$ = $\frac{r_t}{\alpha} - Q_{MF2}(s_{j,t})$. The latter is implemented by multiplying the unchosen model-free Q values by 1- α, as follows: $Q_{MF1}(a_{unchosen,t})$*1- α *and* $Q_{MF2}(s_{unchosen,t})$*1- α. *The choice rule is implemented as follows:*

$$P(a_{i,t}) = \frac{\exp\,[\beta MF *\ Q_{MF1}(a_{i,t}) + \beta MB *\ Q_{MB}(a_{i,t}) + \rho * rep(a_{i,t})]}{\Sigma_{a'}(\exp\,[\beta MF * (\ Q_{MF1}(a'_t)) + \beta MB * (\ Q_{MB}(a'_t)) + \rho * (rep(a'_t)])}$$

Model C: This model is based on Decker, et al. [8] and is identical to Model B, except that it omits the decay of unchosen values and the rescaling of rewards.

Model D: This is identical to model B, without the reward rescaling feature (analogously it is the same as Model C but it includes a decay of unchosen values).

Model E: this is identical to model D with an additional free parameter wherein the decay or 'forgetting' of the value of unselected options is governed by 'αD', rather than 1- α.

Model F: this is identical to model D with rescaling only on reward values (not qt1)

Model G: this is identical to model D with separate learning rates for rewarding and non-rewarding trials.

<u>Group-Level Modelling</u>

For each of the models above, hierarchical Bayesian estimation was carried out using we Markov Chain Monte Carlo (MCMC) techniques (specifically the No-U-Turn variant of Hamiltonian Monte Carlo) as implemented in the Stan modelling language. All parameters were specified as normally distributed; parameters with (0,1) ranges (α1, α2, αD, and ω) were then inverse logit transformed. Means of inverse temperature parameters (β1, βMF, and βMB) were constrained to be greater than 0. Weakly informative priors were used: for means, normal(0,2.5) for α1, α2, αD, ω, and ρ or normal(0,5) for β1, βMF, and βMB; for scales, half-Cauchy(0,1), constrained to be greater than 0, for α1, α2, αD, ω, ρ, and βMF or half-Cauchy(0,2), constrained to be greater than 0, for β1, and β1 MB. For model-selection, we ran four chains of 4,000 samples each, discarding the first 2,000 samples of each chain for burn-in. When modelling the larger dataset, we reduced this to 2,000 samples total for speed.

<u>Model Comparison</u>

To determine the best model, we ran each model on a subset of N=100 of the total sample. We compared models using a range of indicators of validity, with a summary presented in the table below.

(i) MCMC diagnostics

We first examined the chains visually for convergence and also computed Gelman and Rubin's (1992) potential scale reduction factors. For this, large values indicate convergence problems, whereas values near 1 are consistent with convergence. We ensured that these diagnostics were less than 1.05 for all variables. Model A had 78 divergent transitions and Model B had 371. Models C and D had no divergent transitions. Model E had 1 divergent transition and model F had 2.

(ii) LOOIC

We compared the fit of each model using the LOOIC (loo package in R).The lowest LOOIC was found for Model D, followed closely by model E, F then B.

(iii) Posterior Predictive Checks (PPC)

We examined the trial-by-trial predictions for subjects' choices ('y_pred') derived from the model and tested how closely they mirrored actual choices per subject ('y_actual'). For each subject we calculated the proportion of trials where the predicted choice was the same as the actual one and summarised that consistency metric across the entire sample (mean, min, max, and proportion of subjects with consistency >80%). Model G performed the best considering all PPC metrics combined, but there were relatively modest differences across models.

(i) Parameter Recovery

We tested parameter recovery by simulating new choices from agents operating under the true estimated median parameters of our N=100 subjects. We then fitted our models to these choices to test if we could recover the true data generating parameters. We computed correlation coefficients as a measure of parameter recovery. Model B and F showed the strongest recovery of model-based betas at 0.74, but rates were similar at 0.73 for model G, 0.72 for model E, 0.71 for Model D and 0.70 for Model C.

Based on the combination of validation checks, we progressed with Model D for comparison analysis with hierarchical logical regression (HLR) and the point-estimate (PE) approach. Most notably, model D was one of the few models with no divergent transitions, had the lowest LOOIC, and strong performance on all other metrics, if not the best.

Supplementary Method 4. Comparing different estimation approaches

There are various ways to analyse data on this task. Hierarchical logistic regression (HLR) models are a popular choice, but there are alternatives range from extremely computationally cheap Point estimates (PE) to expensive generative models using Hierarchical Bayesian modelling (HB). We tried these alternatives and selected the best approach in a data driven manner, guided by the reliability and external validity of the measures.

Point estimates were calculated by the sum of the probability of staying on [Common, Rewarded trials + Rare, Unrewarded] minus the sum of the probability of staying on [Common, Unrewarded + Rare, Rewarded trials]. Using PE could cut down computational demand and time to calculate estimates, and so may be ideally suited to app-based implementation. However, research suggests they suffer from poor reliability as they fail to consider trial-by-trial individual variability [9]. Both HLR and HB methods account for uncertainty at the level of individual subjects and in the context of the twostep task, have been shown to perform similarly well [4]. For the HB model, we fitted candidate models and selected the best to bring forward for comparison to these other approaches (as described in **Analysis S3**).

We operationalised the (i) internal consistency of the different measures as Cronbach's alpha comparing model-based planning estimates derived from participants' 1st 100 trials to their 2nd 100. We tested this on N=1451 individuals that had complete demographic and mental health data. Note that we did not use odds/evens here, because HB relies on information accumulated slowly over many trials occurring in series. We also tested for (ii) test re-test reliability from a subset of N=423 who completed two sets of 200 trials of Cannon Blast. Finally, we assessed (iii) the external validity of these measures by testing the relationship between estimates of model-based planning and sociodemographic differences N=1451.

As outlined above, we compared three methods of calculating model-based estimates: a point estimate approach (PE), hierarchical logistic regression (HLR) and hierarchical Bayesian modelling (HB). The three analytic approaches produced highly correlated estimates (HLR vs PE: r=.94, HLR vs HB: r=.82, PE vs HB: r=.74).

Using the split-half method where model-based scores from first 100 trials were compared with model-based estimates from the second 100 trials, we found the HLR demonstrated the greatest reliability with fair reliability (r=.56, **Table S18**). Point estimates and computational modelling also demonstrated fair split-half reliability of model-based estimates (PE: r=.48, HB: r=.44). Again, we found the HLR demonstrated the highest test-retest reliability with fair reliability (ICC1=.54). Point estimates also demonstrated fair test retest reliability (ICC1=.63), while Hierarchical Bayesian model showed poor reliability (ICC1=.29). In terms of external

validity, the HLR demonstrated the best signal between model-based planning and individual differences (**Table S19**).

Result**:** HLR outperforms PE and HB across all metrics

Supplementary Notes

Supplementary Note 1. Information Sheet for The Neureka project In-App

The Neureka app comes from a group of scientists at the Global Brain Health Institute who are trying to uncover new ways to detect and prevent disorders of brain health. The brain is a big mystery and we need the help of people like you to solve it.

At Neureka, we think that everyone should be able to participate in science, that's we are taking experiments out of the lab, and into your smartphone. Our team have been hard at work for the past year making games that are not only fun to play, but help us learn about the brain at the same time.

We aim to use your anonymous data, combined with data from other players around the world, to conduct one of the biggest neuroscience studies of all time!

What will happen if I agree to take part?

You will be asked to provide an email-address and a password in order to register an account on the app. We need your email address so that you can sign into the app across different devices and so that we can contact you in future about your contribution to science and any future studies you may want to participate in. You can turn email communication off at any time by changing your settings.

After providing your email you will be asked to complete some basic demographic information and to complete a 'science challenge', where you will play games that tell us things about how your brain works and provide us with some personal information about your physical and mental health, lifestyle and family history. Once complete, you will be free to play the games whenever you like and to complete additional science challenges, at your leisure.

How long will it take to participate?

To complete science challenge 1, it will take approximately 1 hour and it doesn't need to be completed in one go. After that, you can play each game as much or as little as you like. Likewise, you can provide us with as much information about yourself as you like in our 'about me' section. Other science challenges may take longer, with some of them asking you to play the games according to a schedule for days, weeks or even months. These are entirely optional.

What data will I be sharing?

Your email address, the type of device on which you used the app (e.g. iPhone 6), your performance on the games that you choose to play, the demographic information you will be asked to provide during sign-up and any questionnaires that you complete within the app.

What will happen to my data?

We will use the data you provide us from within the app for academic research only. We will share fully anonymized data with other university researchers to help with the collective research effort of improving early detection of dementia and mental health disorders. We will never share personally identifiable information, such as your email address, with anyone. We will publish the findings that your contribution has helped generate in scientific journals and make them available for you to read and share online at www.neureka.ie.

Can I access my data?

You are also free to download all data that you have submitted through the app by tapping "settings"->"GDPR"->"download my data". A password will be required to do this to ensure your privacy and security and data will be sent to your personal email address provided at the time of sign up. Under the Freedom of Information Act 2014, you will have access to any data you provide through the app

for up to 10 years after you have provided it, up until the point at which it has been fully anonymised and we can no longer recover your unique contribution.

Is it confidential?

Yes. We will not disclose any personally identifiable information (e.g. your unencrypted email address) to any third party. However, there are legal limits that apply to confidentiality in the Republic of Ireland, specifically if you provide unsolicited information to the research team pertaining to risk of harm to yourself or another person, this additional information is not protected by confidentiality and may be disclosed to the relevant authorities. Secondly, any information that you provide (solicited or unsolicited) may be disclosed as part of a legal process or police investigation in the Republic of Ireland, without your permission being sought.

Is it secure?

All data you provide through the app is encrypted before it is submitted over the internet. The data you send us through this app is stored and processed in accordance with EU General Data Protection Regulation (GDPR) with our procedures for storing and processing data having undergone and passed review by the data protection officer at Trinity College Dublin.

Gaining credit through SciStarter

We recently partnered with SciStarter, a website that hosts really cool citizen science projects like neureka. If you hold a SciStarter account under the same email as you have registered with neureka, you will automatically receive credit for that on SciStarter. This “linking” of information for registered SciStarter users is done in a fully encrypted way.

Connecting to other studies

Neureka is a powerful tool for engaging people in research, allowing study participants to take part at home and from all over the world. We want to share it with other researchers to help facilitate other important studies, allowing them to compare the same tasks and tools in different populations. If you are part of a study that is using Neureka to gather data, you will be asked by the researchers to enter a Study ID in the settings section of the app. They will provide you with this ID. By providing us with a valid ID, you agree for us to share your data with that research team. The data that we will share with approved studies are specific. That means that only relevant data are shared as outlined in the specific consent you have completed for that study, e.g. scores on a questionnaire, or data from one of our games and never contain any identifiable information (e.g. email address are never shared).

Do I have to take part in the research to use the app?

If you decide that you don’t want to take part in the research then you should go to the “settings”->”GDPR” screen (accessed through the settings button in the top right of the app) and deselect “participate in research”. This will stop any new data from your device being submitted to our server. Your use of the app won’t be affected by this. If you change your mind you can reselect “participate in research” at any time.

How do I withdraw (i.e., get out) of taking part in the research?

Taking part in this research is completely voluntary. You can stop taking part in the research at any time using the process described above to stop sending any new data from your device to our server. If you want to delete data that you have previously submitted to us from the app then you can email the research team by clicking on the “settings ”->”GDPR” and selecting “erase data”. This will send us an email to delete your data and your data will be deleted from the server and our off-line data store held on a password protected computer in Trinity College Dublin. You will be notified by email once we have completed the process of deleting your data. The only time we won’t be able to delete your data is if we have already performed analyses on it and a scientific report/journal article using this data has been submitted and/or if data has already been fully anonymised.

How much Mobile Data (i.e. smartphone access to the internet without wifi) does the app use?

Not very much. The average user will use less than 3 megabytes in total during their time using the app. This doesn’t include the data used to download or update the app.

Are there any risks involved in participating?

The risks associated with participating in this research are minimal. However, it is possible that you might find the sensitive nature of some of the questions in questionnaires that you might choose to complete upsetting. If you experience upset or distress we encourage you to contact your General Practitioner/Doctor. We also provide information on support services in a dedicated tab within the app.

Are there any benefits to participating?

This study will not benefit you in any direct way. The data that we collect from you will hopefully allow us to develop a tool to better detect early cognitive changes that are markers of future risk of developing dementia and mental health disorders and in this way your contribution might benefit others.

Will the research team take responsibility for diagnosing app users with dementia or mental health disorders?

No. The games within the app are not diagnostic in isolation and your basic performance level is not indicative of risk for disorders of brain health. Users will not be provided with any processed scores, or individualised predictions on risk of dementia or mental health disorders based on the data that they provide through the app. While we hope that one day, some combination of these measures might help us to detect illness early – the science is simply not there yet.

Who is running this study?

The study is being conducted by a research team based at the Global Brain Health Institute at Trinity College Dublin in the Republic of Ireland. The study has received ethical approval from the School of Psychology Research Ethics Committee at Trinity College Dublin.

In taking part and providing consent, you are agreeing to participate in a study that handles your data in line with the Republic of Ireland Data Protection Act 2018 which is in line with the EU General Data Protection Regulation (GDPR) 2016/679. For information on Data Protection law in Ireland see https://dataprotection.ie/docs/A-guide-to-your-rights-Plain-English-Version/r/858.htm

Supplementary Figures/Tables

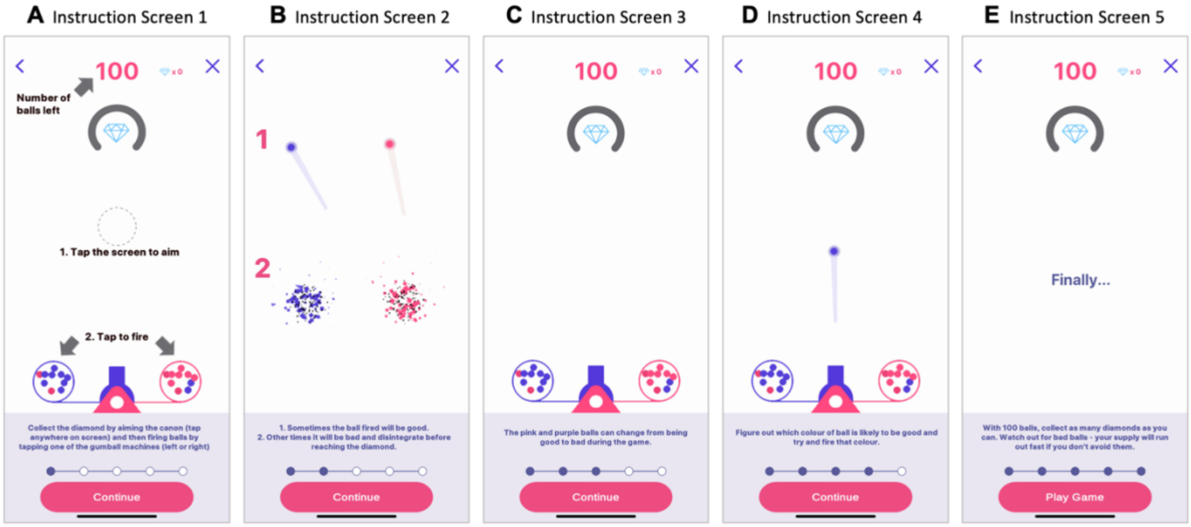

Figure S1. Passive instructions from Cannon Blast shown to participants.
A. Instructs participants of the aim of Cannon Blast (i.e., to collect diamonds). Demonstrates visually the steps a participant must take: first tapping the screen to aim the cannon and second to tap a coloured container to load a ball. **B**. Participants are made aware that they will either be rewarded with a good ball or unrewarded with a bad ball that disintegrates upon firing. **C**. Participants are told of the drifting nature of the ball quality over the course of the game. **D**. Participants are instructed to figure out which ball colour is likely to be rewarding and to select this colour. **E**. Participants are explicitly told of the finite source of balls (100) and to collect as many diamonds as possible by trying to avoid bad balls.

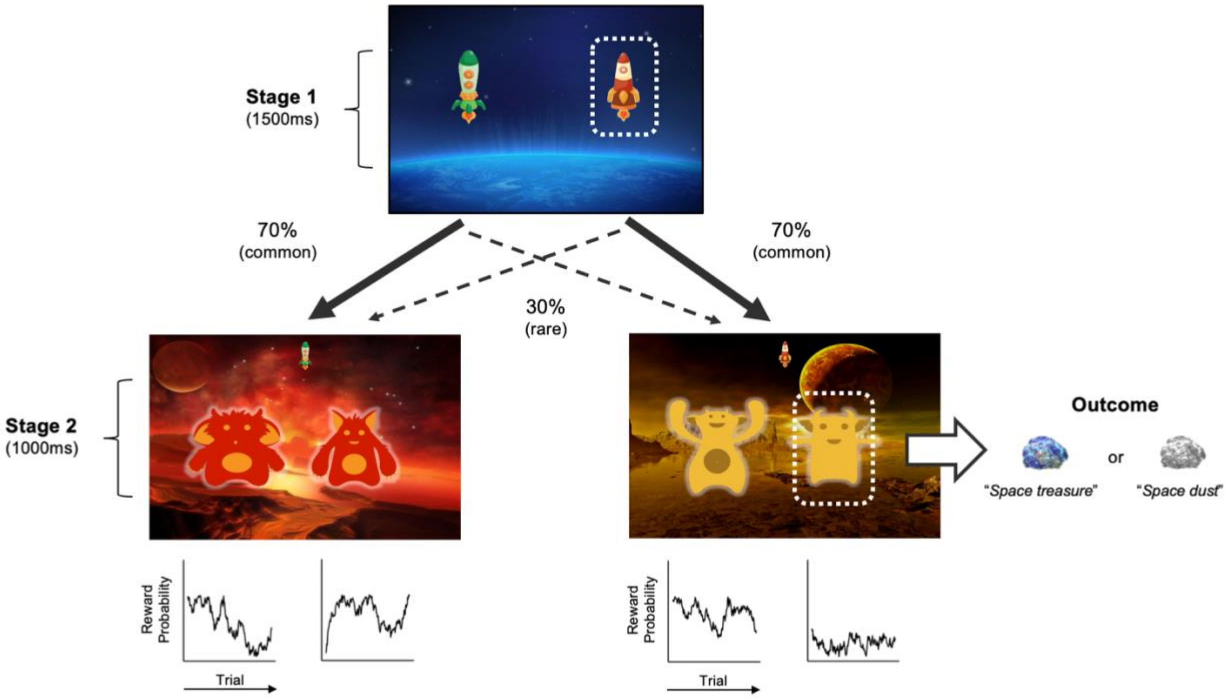

Figure S2. Two-step reinforcement learning task for assessing model-based learning. This paradigm (adapted by [8]) consisted of two stages where in the first stage subjects chose between two rockets which probabilistically transitioned to one of two second stage planets. Each rocket travelled to a preferred planet 70% of the time ('common' transition) or to the alternative planet 30% of the time ('rare' transition). In the second stage, subjects had to choose between two aliens, both with a unique probability of being rewarding ('space treasure') or unrewarding ('space dust') that drifted slowly and independently over the course of the experiment in pre-determined trajectories. Participants had to simultaneously track the distinct reward probabilities of each alien and incorporate knowledge of the transition structure in order to maximise their chance of reward.

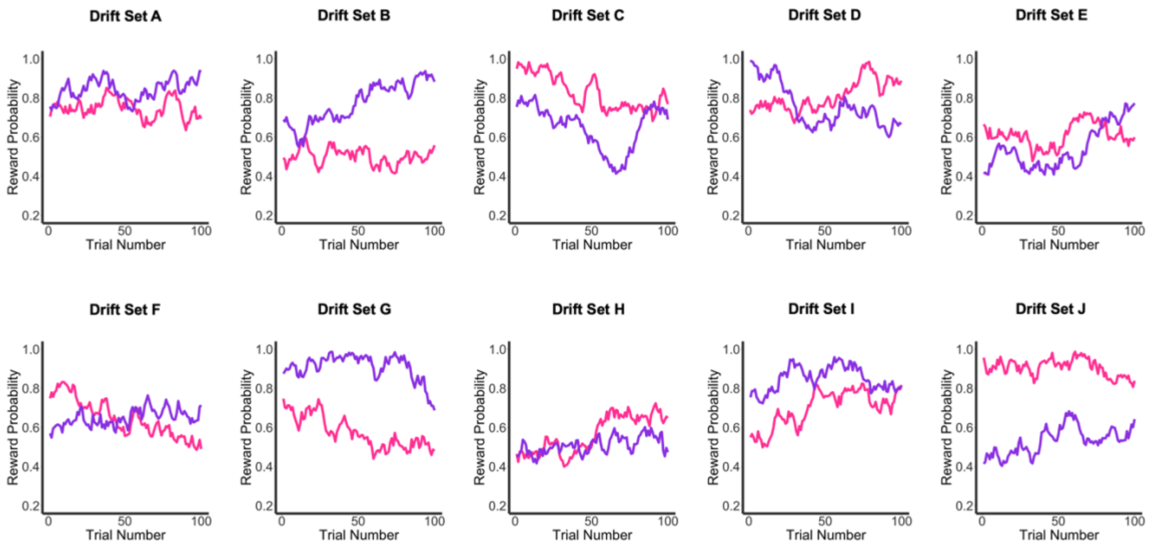

Figure S3. Reward trajectories for each of the ten drifting reward probabilities sets. On participants first play ('Risk Factors') participants were randomly assigned to receive either Drift Set A or Drift Set B at each block of trials. On participants repeated plays ('Free Play'), participants were randomly assigned to receive one out of the possible ten drifts (A – J).

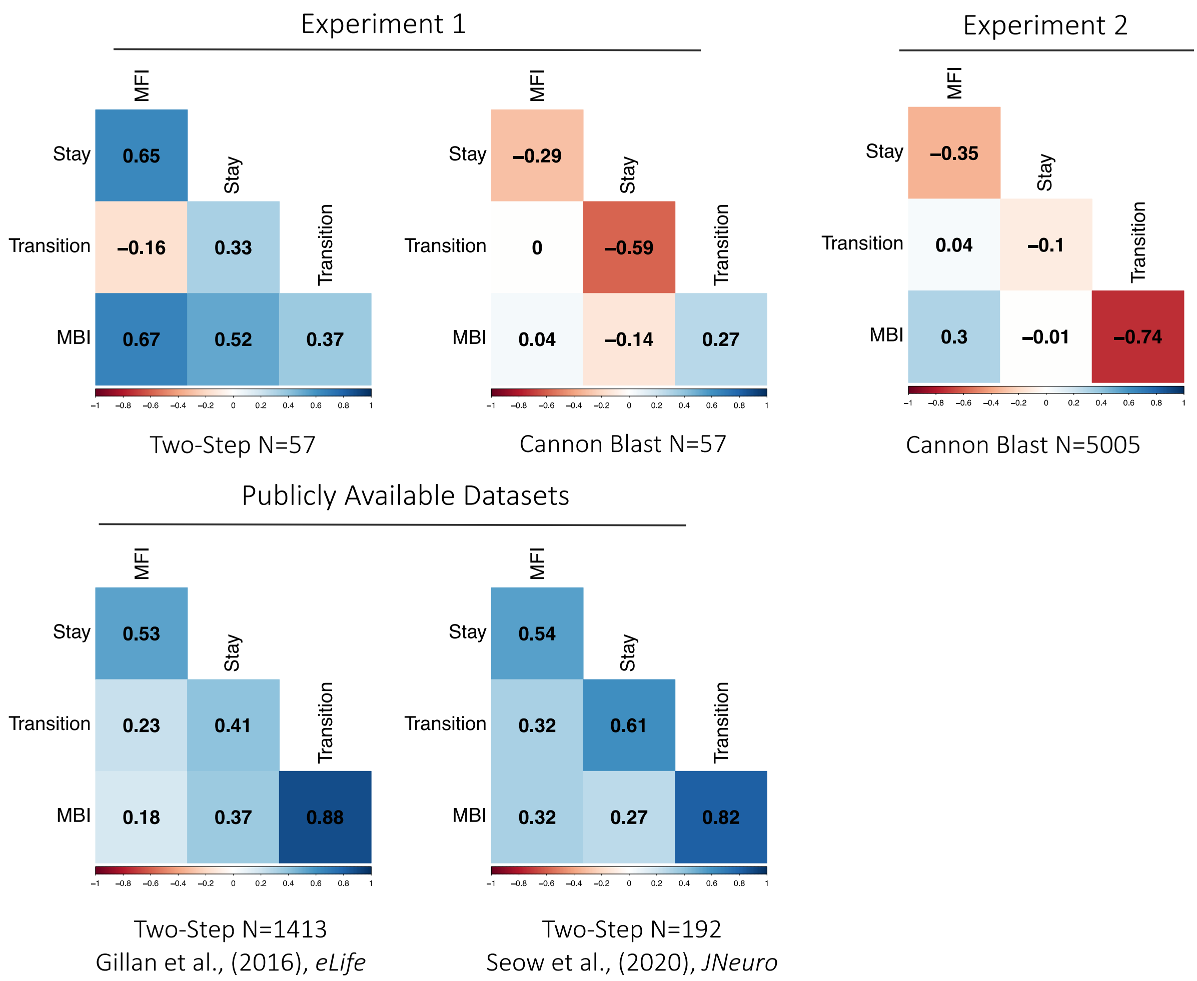

Figure S4. Within-task correlations in our dataset and other publicly available datasets.
Within-task correlations of task outcomes including choice preservation ('Stay'), transition, model-based (MBI) and model-free (MFI) in our data sets (top graphs) and in publicly available datasets (bottom graphs). There are differences between the within-task correlations from our dataset to other data sets using the classic two-step task i.e., the correlation between MBI and MFI. Within task correlations in our dataset from Cannon Blast are replicated in the larger citizen science sample.

Supplementary Tables

Table S1. Task Parameters used in Traditional Two-step Task and Cannon Blast

	**Traditional Task**	**Cannon Blast**
Trial Number	200	200
Choice Time	1500ms	Unlimited
Reward Probability (Mean)	.52	.72[a]
Rewards Received (Mean, Range)	104.32 (83-127)	145.05 (124-161)[b]
Unique Drifts	1	4
Transition Probabilities	70:30	80:20
Pre-task tutorial	40 active training trials	5-step passive instruction
Stated Objective	Collect Space Treasure	Collect diamonds

[a] This is the average reward probability across the two ball colours across all four drift sets in Cannon Blast.
[b] This is the average rewards actually received by participants across all four drift sets in Cannon Blast.

Table S2. Descriptive Information of Easy and Medium Levels of Difficulty in Cannon Blast

Difficulty	Level	Description	Diamond Behaviour	Hit Rates
Easy	1	Static diamond	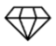	83%
Easy	2	Moving diamond horizontally or vertically	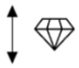 or 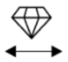	53%
Easy	3	Moving diamond in direction of an arc	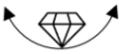	44%
Easy	4	Static diamond facing down in 3 directions	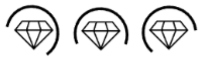	29%
Medium	5	Static diamond with shell facing down	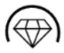	75%
Medium	6	Moving diamond diagonally with shell facing down	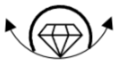	20%
Medium	7	Static diamonds with rotating shells	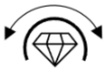	38%
Medium	8	Static diamonds with gates	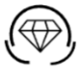	45%

Table S3. Demographic Characteristics in Experiment 2 in the Whole Sample and the two Age, Gender and Education Matched Samples.

Characteristics	Whole Sample	80:20 Matched	70:30 Matched
N	5005	2138	2138
		**Mean (SD)**	
Age	45.69 (14.54)	47.23 (13.62)	48.12 (14.42)
		**N (% of N)**	
Gender			
Cisgender woman	3225 (64%)	1397 (65%)	1429 (67%)
Cisgender man	1683 (34%)	701 (33%)	661 (31%)
Non-cisgender[a]	82 (1.9%)	37 (1.9%)	44 (1.9%)
Preferred not to say	15 (>.01%)	3 (>.01%)	4 (>.01%)
Education Attainment			
No formal education	71 (1%)	21 (1%)	25 (1%)
Lower Second Level	399 (8%)	161 (8%)	167 (8%)
Upper Second Level	1314 (26%)	579 (27%)	594 (27%)
University/College	2043 (42%)	846 (39%)	876 (41%)
Master’s	958 (19%)	434 (20%)	377 (18%)
PhD (or equivalent)	219 (4%)	97 (5%)	99 (5%)

[a]‘Non-cisgender’ includes those who identify as Transgender Man, Transgender Woman, Non-Binary, or not-listed
SD=Standard deviation

Table S4. Mental Health Summary in Experiment 2 (N=1451): Mean symptoms and their association with age, gender, and education.

			Age		Gender		Education	
**Construct (Measure)**	**a**	**Mean (SD)**	**r**	**p**	**diff[a]**	**p**	**F[b]**	**p**
Apathy (AES)	.90	31.54 (8.55)	-.23	<.001***	1.08	.119	39.51	<.001***
Alcohol Dependency (AUDIT)	.83	4.16 (4.28)	-.14	<.001***	1.23	.000**	6.55	.001**
Impulsivity (BIS)	.87	57.98 (11.56)	-.25	<.001***	-.52	.859	36.94	<.001***
Eating Disorders (EAT-26)	.87	29.27 (15.94)	-.16	<.001***	-5.21	<.001***	1.82	.162
Social Anxiety (LSAS)	.95	28.65 (15.31)	-.27	<.001***	-3.49	<.001***	27.97	<.001***
OCD (OCI-R)	.91	13.94 (11.34)	-.30	<.001***	-.47	.890	11.79	<.001***
Schizotypy (SCZ)	.89	12.68 (8.00)	-.40	<.001***	-1.08	.083	31.05	<.001***
Depression (SDS)	.90	38.91 (10.74)	-.35	<.001***	-3.69	<.001***	42.12	<.001***
Trait Anxiety (STAI)	.95	42.40 (13.62)	-.41	<.001***	-3.27	<.001***	23.95	<.001***
**Transdiagnostic Factor**		**Mean (SD)**	**r**	**p**	**diff[a]**	**p**	**F[b]**	**p**
Anxious-Depression	-	2.92 (.89)	-.34	<.001***	-.11	.128	42.28	<.001***
Compulsivity and Intrusive Thought	-	1.45 (.94)	-.38	<.001***	-.23	<.001***	7.11	<.001***
Social Withdrawal	-	1.50 (.94)	-.25	<.001***	-.23	<.001***	19.14	<.001***

AES: Apathy Evaluation Scale, AUDIT: Alcohol Use Disorders Identification Test, BIS: Barratt's Impulsivity Scale, EAT: Eating Attitudes Test, LSAS: Liebowitz Social Anxiety Scale, OCD: Obsessive Compulsive Disorder, OCIR: Obsessive Compulsive Inventory Revised, SCZ: Short Scale Measures for Schizotypy, SDS: Self-rated Depression Scale, STAI: Spielberger's Trait Anxiety Inventory.
a: Cronbach's alpha
SD=Standard deviation
[a] Mean group difference between cis-gender men and cis-gender women. Positive values indicate higher scores among men, while negative t-values indicate higher scores in women.
[b] Positive F values indicate higher scores among those who did not attain third level education compared with those who obtained third level education and those who attained greater than third level education.

Table S5. Descriptive Information of those with Repeated Plays of Cannon Blast

Number of participants who played Free Play section	785
Number of Free Play sessions completed	2929
Level of difficulty chosen	
Easy	1045
Medium	1161
Hard	723
Mean number of free play sessions (SD)	3.73 (9.11)
Median number of free play sessions	2
Range of free play sessions (Min.-Max.)	1 – 138
Mean time (in days) between 1st play and follow-up (SD)	4.46 (5.86)
Median of time (in days) elapsed	2
Range of time (in days) elapsed	0-30

Table S6. Descriptive Information of Reward Drift Sets assigned at Each Block

Reward Drift Set	Mean Reward Probability		Mean Reward Probability	Mean Reward Difference	SD Reward Probability		Mean SD Reward	Mean SD Difference
	Purple	Pink			Purple	Pink		
**A**	.845	.748	.797	.096	.053	.049	.052	.005
**B**	.774	.501	.638	.273	.103	.041	.072	.062
**C**	.649	.829	.739	.180	.113	.088	.101	.025
**D**	.756	.809	.783	.053	.109	.075	.092	.034
**E**	.542	.611	.576	.069	.110	.059	.084	.051
**F**	.649	.647	.648	.002	.043	.096	.070	.053
**G**	.909	.575	.742	.335	.066	.081	.074	.015
**H**	.510	.564	.537	.054	.043	.096	.069	.053
**I**	.849	.698	.774	.151	.062	.092	.077	.030
**J**	.532	.906	.719	.374	.069	.043	.056	.026

Only Drift Set A & Drift Set B used in participants first play (‘Risk Factors’ section of the app). All ten drifts (A-J) used in repeated plays of Cannon Blast (‘Free Play section of the app)
Mean reward/SD difference=Absolute value between the highest mean reward/SD probability minus the lowest mean reward/SD probability

Table S7. Logistic Regression Analysis across various Task Modifications

	**β (SE)**	**z value**	**p value**
Base Model N=5005			
Intercept	1.38 (.01)	111.52	<.001***
Reward	.47 (.01)	53.24	<.001***
Transition	-.08 (.00)	-18.37	<.001***
Reward: Transition	.27 (.01)	39.84	<.001***
80:20 Transition N=2138[a]			
Intercept	1.31 (.02)	72.78	<.001***
Reward	.47 (.01)	34.67	<.001***
Transition	-.09 (.01)	-12.79	<.001***
Reward: Transition	.31 (.01)	28.69	<.001***
70:30 Transition, N=2138 [a]			
Intercept	1.33 (.02)	72.77	<.001***
Reward	.44 (.02)	33.66	<.001***
Transition	-.07 (.01)	-11.36	<.001***
Reward: Transition	.22 (.01)	23.14	<.001***
Easy-1st Block, N=5005			
Intercept	1.24 (.01)	97.67	<.001***
Reward	.44 (.01)	46.59	<.001***
Transition	-.09 (.01)	-15.94	<.001***
Reward: Transition	.31 (.01)	39.72	<.001***
Medium-2nd Block, N=5005			
Intercept	1.55 (.02)	93.98	<.001***
Reward	.48 (.01)	42.79	<.001***
Transition	-.07 (.01)	-11.38	<.001***
Reward: Transition	.23 (.01)	27.27	<.001***
Drift A-1st Block, N=2395			
Intercept	1.29 (.02)	67.08	<.001***
Reward	.45 (.02)	28.54	<.001***
Transition	-.09 (.01)	-9.95	<.001***
Reward: Transition	.28 (.01)	23.28	<.001***
Drift B-1st Block, N=2610			
Intercept	1.19 (.02)	70.43	<.001***
Reward	.44 (.01)	37.28	<.001***
Transition	-.08 (.01)	-11.48	<.001***
Reward: Transition	.34 (.01)	31.99	<.001***

* p<.05, ** p<.01, *** p<.001
SE=standard error
[a] Subsample of total dataset to achieve two age, gender and education matched samples
Dependent variable ‘Stay’ coded as (1,0)
Independent variables coded as: Reward (-1,1: Non-Rewarded, Rewarded); Transition (-1,1: Rare, Common)

Table S8. Associations between Model-based Planning and Individual Differences (N=5005) and Clinical Associations (N=1451)

	β (SE)	z value	p value
Individual Differences, N=5005			
Age	-.02 (.00)	-4.39	<.001***
Gender	-.02 (.00)	-3.34	.001**
Education	-.04 (.00)	-9.41	<.001***
Questionnaire Total Scores[a], N=1451			
Alcohol Dependency	.01 (.01)	.71	.478
Apathy	.01 (.01)	.69	.492
Depression	-.02 (.01)	-1.88	.061
Eating Disorder	-.02 (.01)	-2.17	.031*
Impulsivity	-.02 (.01)	-2.22	.026**
OCD	-.01 (.01)	-1.62	.105
Schizotypy	.01 (.01)	.58	.564
Social Anxiety	.00 (.01)	.26	.797
Trait Anxiety	-.00 (.01)	-.14	.888
Transdiagnostic Factors[b], N=1451			
Anxious-Depression	-.00 (.01)	-.34	.733
Compulsivity	-.03 (.01)	-2.87	.004**
Social Withdrawal	.02 (.01)	1.62	.106

* p<.05, ** p<.01, *** p<.001

SE=standard error, OCD=Obsessive Compulsive Disorder

[a] Independent models controlling for age gender and education

[b] Covariate model controlling for age, gender and education

Table S9. Associations with Model-free, Model-based, Stay and Transition estimates from Cannon Blast with Individual Differences and Clinical Associations

	β(SE)	z value	p value
Individual differences, N=5005			
Stay			
Age	-.01 (.01)	-.90	.368
Gender	.02 (.02)	1.57	.117
Education	-.00 (.01)	.00	.997
Model-Free			
Age	-.08 (.01)	-5.61	<.001***
Gender	-.09 (.01)	-6.28	<.001***
Education	-.10 (.01)	-6.85	<.001***
Transition			
Age	.03 (.01)	-2.44	<.001***
Gender	-.00 (.02)	-.19	.852
Education	.09 (.01)	6.17	<.001***
Model-based			
Age	-.06 (.01)	-4.39	<.001***
Gender	-.05 (.01)	-3.34	.001**
Education	-.13 (.01)	-9.41	<.001***
Clinical differences, N=1451			
Stay			
Anxious Depression	.01 (.03)	.40	.686
Compulsivity	.03 (.03)	1.13	.260
Social Withdrawal	.02 (.03)	.57	.566
Model-Free			
Anxious Depression	-.01 (.03)	-.34	.736
Compulsivity	-.10 (.03)	-3.28	.001**
Social Withdrawal	.02 (.03)	.55	.582
Transition			
Anxious Depression	.02 (.03)	.71	.478
Compulsivity	.06 (.03)	2.16	.031*
Social Withdrawal	-.05 (.03)	-1.57	.118
Model-based			
Anxious Depression	-.01 (.03)	-4.15	.733
Compulsivity	-.08 (.06)	-2.88	.004**
Social Withdrawal	.05 (.03)	1.62	.106

* p<.05, ** p<.01, *** p<.001
SE=standard error
Gender: Comparison of cisgender woman with reference to cisgender man
Education: negative beta values reflect those with less education associated with greater MBI deficits
Covariate model controlling for age, gender and education

Table S10. Linear Regressions Predicting Stay Behaviour by Reward, Transition and Diamond Hit on the Previous Trial.

	β(SE)	z value	p value
Intercept	1.38 (.03)	49.33	<.001***
Reward	.43 (.03)	16.22	<.001***
Transition	.01 (.03)	.42	.676
Diamond Hit	-.05 (.03)	-1.94	.053
Reward: Transition	.19 (.03)	7.22	<.001***
Reward: Diamond Hit	-.14 (.03)	-5.39	<.001***
Transition: Diamond Hit	.08 (.03)	3.10	.002**
Reward: Transition: Diamond Hit	-.09 (.03)	-3.35	<.001***

* p<.05, ** p<.01, *** p<.001
SE=standard error
Dependent variable 'Stay' coded as (1,0)
Independent variables coded as: Reward (-1,1: Non-Rewarded, Rewarded); Transition (-1,1: Rare, Common); Diamond Hit (-1,1: Miss, Hit)

Table S11. Linear Regressions assessing the External Validity across various Task Modifications

	β (SE)	z value	p value
80:20 Transition, N=1020			
CIT	-.01 (.01)	-.89	.374
Age	-.03 (.01)	-2.99	.003**
Gender	-.04 (.01)	-23.29	.001**
Education	-.04 (.01)	-4.18	<.001***
70:30 Transition, N=1051			
CIT	-.05 (.01)	-4.43	<.001***
Age	-.05 (.01)	-3.92	<.001***
Gender	.00 (.01)	.40	.689
Education	-.04 (.01)	-4.12	<.001***
Easy-1st Block, N=2369			
CIT	-.03 (.01)	-3.82	<.001***
Age	-.04 (.01)	-6.24	<.001***
Gender	-.01 (.01)	-1.55	.122
Education	-.05 (.01)	-7.08	<.001***
Medium-2nd Block, N=2369			
CIT	-.02 (.01)	-3.39	.001**
Age	-.03 (.01)	-4.31	<.001***
Gender	-.01 (.01)	-1.38	.167
Education	-.03 (.01)	-5.46	<.001***
Drift A-1st Block, N=1135			
CIT	-.05 (.01)	-4.94	<.001***
Age	-.06 (.01)	-5.99	<.001***
Gender	-.01 (.01)	-.68	.500
Education	-.05 (.01)	-4.88	<.001***
Drift B-1st Block, N=1234			
CIT	-.01 (.01)	-.61	.540
Age	-.03 (.01)	-2.75	.006**
Gender	-.01 (.01)	-1.42	.155
Education	-.05 (.01)	-5.24	<.001***

* p<.05, ** p<.01, *** p<.001
CIT=Compulsivity
Linear regression models: model-based index ~ CIT + age + gender + education
Gender: Comparison of cisgender woman with reference to cisgender man
Education: negative beta values reflect those with less education associated with greater MBI deficits

Table S12. Full Comparison Models across Task Modifications

	β (SE)	z value	p value
Transition Ratio, N=2071			
CIT	-.03 (.01)	-3.62	<.001***
Age	-.04 (.01)	-4.86	<.001***
Gender	-.02 (.01)	-2.05	.041*
Education	-.04 (.01)	-5.87	<.001***
Transition ratio	-.01 (.01)	-.32	.748
CIT*Transition ratio	-.02 (.01)	-2.29	.022*
Age*Transition ratio	-.00 (.01)	-.45	.658
Gender*Transition ratio	.02 (.01)	2.61	.009**
Education*Transition ratio	.00 (.01)	.16	.874
Difficulty/Order, N=2369			
CIT	-.02 (.01)	-4.12	<.001***
Age	-.04 (.01)	-6.05	<.001***
Gender	-.01 (.01)	-1.67	.095
Education	-.04 (.01)	-7.18	<.001***
Difficulty/Order	-.12 (.04)	-3.13	.002**
CIT*Difficulty/Order	.00 (.00)	.71	.476
Age* Difficulty/Order	.01 (.00)	2.40	.017*
Gender* Difficulty/Order	.00 (.00)	.28	.782
Education* Difficulty/Order	.01 (.00)	2.15	.032*
Reward Drift Set, N=2369			
CIT	-.03 (.01)	-3.94	<.001***
Age	-.04 (.01)	-6.16	<.001***
Gender	-.01 (.01)	-1.48	.138
Education	-.05 (.01)	-7.14	.002**
Drift set	.05 (.08)	.65	.518
CIT*Drift set	.02 (.01)	-3.09	.002**
Age*Drift set	.02 (.01)	2.25	.024*
Gender*Drift set	-.00 (.01)	-.53	.595
Education*Drift set	-.00 (.01)	-.19	.853

* p<.05, ** p<.01, *** p<.001
CIT=Compulsivity
Full comparison models: model-based index ~ (CIT + age + gender + education)*[task parameter]
Task parameters coded in model as: Transition ratio (-1,1: 80:20, 70:30); Difficulty/Order (-1,1: Easy-1st Block, Medium-2nd Block); Drift set (-1, 1: Drift A, Drift B)
Gender: Comparison of cisgender woman with reference to cisgender man
Education: negative beta values reflect those with less education associated with greater MBI deficits

Table S13. Intraclass Correlation Co-efficient (ICC) testing the Test-retest Reliability of Model-based Indices across Task Difficulty/Order

	N	ICC_1	CI Lower	CI Upper
1st play Easy vs 2nd play Easy	689	.35	.30	.41
1st play Easy vs 1st play Medium	556	.49	.44	.54
1st play Medium vs 2nd play Easy	689	.45	.40	.50
1st play Medium vs 2nd play Medium	556	.51	.46	.56

ICC=Intraclass Correlation Co-efficient, CI=Confidence Interval

Table S14. Assessing the Impact of Increasing Trial Number per Participant on Mean-level Model-based Indices and their Reliability (N=716)

Trials	M (SD)	Range	Split-half [95% CI]
25	.35 (.33)	[-.63-1.20]	.41 [.35-.47]
50	.33 (.33)	[-.52-1.55]	.51 [.46-.57]
75	.31 (.32)	[-.58-1.61]	.53 [.47-.58]
100	.31 (.33)	[-.50-1.79]	.59 [.54-.64]
125	.30 (.33)	[-.46-1.83]	.63 [.58-.67]
150	.28 (.33)	[-.35-1.97]	.64 [.59-.68]
175	.28 (.34)	[-.38-2.14]	.66 [.62-.70]
200	.28 (.34)	[-.45-2.25]	.68 [.64-.72]
225	.27 (.35)	[-.37-2.28]	.69 [.65-.72]
250	.27 (.33)	[-.25-2.25]	.70 [.66-.73]
275	.26 (.24)	[-.30-2.31]	.71 [.67-.75]
300	.26 (.34)	[-.27-2.31]	.71 [.68-.75]

M=Mean, SD=Standard Deviation, CI=Confidence Interval
Split-half reliability co-efficient assessed using odd-evens approach

Table S15. Logistic Regression Analysis with Increasing Trial Number (N=716)

	β (SE)	z value	p value
**25 Trials**			
Intercept	.96 (.04)	25.51	<.001***
Reward	.41 (.03)	13.65	<.001***
Transition	-.11 (.03)	-4.13	<.001***
Reward: Transition	.37 (.03)	12.05	<.001***
**50 Trials**			
Intercept	1.06 (.03)	31.12	<.001***
Reward	.42 (.03)	16.25	<.001***
Transition	-.10 (.02)	-5.34	<.001***
Reward: Transition	.33 (.02)	13.52	<.001***
**75 Trials**			
Intercept	1.11 (.03)	34.78	<.001***
Reward	.44 (.02)	18.01	<.001***
Transition	-.07 (.02)	-4.51	<.001***
Reward: Transition	.32 (.02)	14.72	<.001***
**100 Trials**			
Intercept	1.16 (.03)	36.62	<.001***
Reward	.49 (.02)	19.11	<.001***
Transition	-.07 (.01)	-4.72	<.001***
Reward: Transition	.32 (.02)	15.62	<.001***
**125 Trials**			
Intercept	1.16 (.03)	38.11	<.001***
Reward	.46 (.02)	19.95	<.001***
Transition	-.06 (.01)	-4.95	<.001***
Reward: Transition	.30 (.02)	15.98	<.001***
**150 Trials**			
Intercept	1.19 (.03)	39.79	<.001***
Reward	.47 (.02)	19.95	<.001***
Transition	-.06 (.01)	-5.41	<.001***
Reward: Transition	.29 (.02)	15.81	<.001***
**175 Trials**			
Intercept	1.22 (.03)	40.74	<.001***
Reward	.47 (.02)	20.87	<.001***
Transition	-.08 (.01)	-6.65	<.001***
Reward: Transition	.28 (.02)	15.73	<.001***
**200 trials**			
Intercept	1.25 (.03)	41.51	<.001***
Reward	.48 (.02)	21.45	<.001***
Transition	-.09 (.01)	-7.12	<.001***
Reward: Transition	.28 (.02)	15.91	<.001***
**225 trials**			
Intercept	1.23 (.03)	43.52	<.001***
Reward	.48 (.02)	22.64	<.001***
Transition	-.07 (.01)	-6.94	<.001***
Reward: Transition	.28 (.02)	16.37	<.001***
**250 trials**			

Intercept	1.23 (.03)	45.33	<.001***
Reward	.49 (.02)	24.21	<.001***
Transition	-.06 (.01)	-8.00	<.001***
Reward: Transition	.27 (.02)	16.74	<.001***
**275 trials**			
Intercept	1.24 (.03)	46.34	<.001***
Reward	.48 (.02)	24.07	<.001***
Transition	-.07 (.01)	-7.37	<.001***
Reward: Transition	.26 (.02)	16.12	<.001***
**300 trials**			
Intercept	1.24 (.03)	46.95	<.001***
Reward	.48 (.02)	24.53	<.001***
Transition	-.07 (.01)	-7.38	<.001***
Reward: Transition	.26 (.02)	16.24	<.001***

* p<.05, ** p<.01, *** p<.001
SE=standard error
Dependent variable ‘Stay’ coded as (1,0)
Independent variables coded as: Reward (1,-1: Rewarded, Non-Rewarded); Transition (1,-1: Common, Rare)

Table S16. Linear Regressions assessing the External Validity with Increasing Trial Number (N=716)

	β (SE)	z value	p value
**25 Trials**			
CIT	-.04 (.01)	-3.14	.002**
Age	-.02 (.01)	-1.28	.229
Gender	-.03 (.01)	-2.14	.033*
Education	-.05 (.01)	-3.97	<.001***
**50 Trials**			
CIT	-.04 (.01)	-3.15	.002**
Age	-.03 (.01)	-1.93	.052
Gender	-.02 (.01)	-1.33	.183
Education	-.06 (.01)	-4.87	<.001***
**75 Trials**			
CIT	-.05 (.01)	-3.93	<.001***
Age	-.03 (.01)	-2.23	.026*
Gender	-.00 (.01)	-0.30	.763
Education	-.06 (.01)	-4.82	<.001***
**100 Trials**			
CIT	-.05 (.01)	-3.96	<.001***
Age	-.03 (.01)	-2.62	.026*
Gender	-.00 (.01)	-0.16	.763
Education	-.06 (.01)	-5.03	<.001***
**125 Trials**			
CIT	-.05 (.01)	-3.79	<.001***
Age	-.03 (.01)	-2.42	.016*
Gender	-.01 (.01)	-0.54	.588
Education	-.06 (.01)	-4.80	<.001***
**150 Trials**			
CIT	-.04 (.01)	-3.38	<.001***
Age	-.03 (.01)	-2.29	.022*
Gender	-.01 (.01)	-0.49	.625
Education	-.06 (.01)	-4.93	<.001***
**175 Trials**			
CIT	-.04 (.01)	-3.36	.001**
Age	-.03 (.01)	-2.24	.025*
Gender	-.01 (.01)	-0.40	.689
Education	-.06 (.01)	-4.79	<.001***
**200 trials**			
CIT	-.04 (.01)	-3.32	.001**
Age	-.03 (.01)	-2.10	.036*
Gender	-.01 (.01)	-0.66	.507
Education	-.06 (.01)	-4.71	<.001***
**225 trials**			
CIT	-.05 (.01)	-3.44	.001**
Age	-.03 (.01)	-2.32	.020*
Gender	-.00 (.01)	-0.26	.793
Education	-.06 (.01)	-4.90	<.001***

**250 trials**			
CIT	-.04 (.01)	-3.33	.001**
Age	-.03 (.01)	-2.26	.024*
Gender	-.00 (.01)	-0.24	.809
Education	-.06 (.01)	-4.74	<.001***
**275 trials**			
CIT	-.05 (.01)	-3.49	.001**
Age	-.03 (.01)	-2.44	.020*
Gender	-.00 (.01)	-0.04	.793
Education	-.06 (.01)	-5.09	<.001***
**300 trials**			
CIT	-.04 (.01)	-3.37	.001**
Age	-.03 (.01)	-2.52	.012*
Gender	-.00 (.01)	-0.01	.994
Education	-.06 (.01)	-5.13	<.001***

* p<.05, ** p<.01, *** p<.001
SE=standard error, CIT=Compulsivity
Regression models: model-based index ~ CIT + age + gender + education
Gender: Comparison of cisgender woman with reference to cisgender man
Education: negative beta values reflect those with less education associated with greater MBI deficits
Shaded rows indicate the maximum association observed for each individual difference measure

Table S17. Full Comparison Models with Increasing Trial Number (N=716)

	**β (SE)**	**z value**	**p value**
CIT	-.04 (.01)	-3.60	<.001
Age	-.02 (.01)	-1.88	.061
Gender	-.02 (.01)	-1.39	.165
Education	-.05 (.01)	-4.61	<.001
Trial Number	-.02 (.01)	-2.86	.004
CIT: Trial Number	-.00 (.00)	-0.23	.819
Age: Trial Number	-.01 (.00)	-2.00	.044
Gender: Trial Number	.00 (.00)	3.55	<.001
Education: Trial Number	.00 (.00)	-2.03	.042

* p<.05, ** p<.01, *** p<.001
SE=standard error, CIT=Compulsivity
Regression models: model-based index ~ (CIT + age + gender + education)*Trial Number
Gender: Comparison of cisgender woman with reference to cisgender man
Education: negative beta values reflect those with less education associated with greater MBI deficits

Table S18. Candidate Model Comparison and Evaluation for Hierarchical Bayesian Models

**Model Name**	**A**	**B**	**C**	**D**	**E**	**F**	**G**
**MCMC diagnostics**							
Divergence	78	371	0	0	1	2	0
All rhats <1.05	NO	YES	YES	YES	YES	YES	YES
**LOOIC**	17283	16637	17310	16486	16495	16565	16533
**PPC**							
Mean % agree	0.81	0.82	0.81	0.82	0.82	0.82	0.82
Median % agree	0.82	0.84	0.82	0.84	0.84	0.84	0.84
Prop. over 80% agree	0.59	0.66	0.59	0.63	0.64	0.64	0.60
Min	0.59	0.53	0.60	0.60	0.58	0.59	0.64
Max	0.96	0.96	0.96	0.96	0.96	0.96	0.97
**Parameter Recovery**							
ρ Perseveration	0.96	0.96	0.96	0.97	0.97	0.97	0.97
α1 1st stage LR	0.73	0.82	0.79	0.87	0.86	0.79	-
α2 2nd stage LR	0.64	-	-	-	-	-	-
β1 Inverse temp.	0.74	-	-	-	-	-	-
ω MB/MF weight	0.63	-	-	-	-	-	-
βMB Model-based β	-	0.74	0.70	0.71	0.72	0.74	0.73
βMF Model-free β	-	0.78	0.65	0.75	0.78	0.82	0.81
αD Decay rate	-	-	-	-	0.35	-	-
α+ 1st stage LR+	-	-	-	-	-	-	0.75
α- 1st stage LR-	-	-	-	-	-	-	0.79

All models were run on n=100 subjects (*simulated agents* for parameter recovery), with models estimated from 4K iterations. For each row, the best performing model (or joint-best) is highlighted in green.

Table S19. Comparing the Association between Model-based Index and Individual Differences using Different Analytic Approaches to Calculate Model-based Index

	N=1451				N=423
	**β (SE)**	**z value**	**p value**	**Split-Half (95% CI)**	**Test Retest (95% CI)**
Point Estimates				.48 (.44-.52)	.58 (.52-.63)
CIT	-.07 (.03)	-2.33	.020		
Age	-.10 (.03)	-3.43	.001		
Gender	-.06 (.03)	-2.03	.042		
Education	-.11 (.03)	-4.36	<.001		
Hierarchical Linear Regression				.56 (.51-.58)	.63 (.57-.67)
CIT	-.07 (.03)	-2.51	.012		
Age	-.12 (.03)	-4.40	<.001		
Gender	-.07 (.03)	-2.41	.016		
Education	-.13 (.03)	-5.23	<.001		
Hierarchical Bayesian Model				.44 (.40-.48)	.29 (.21-.36)
CIT	-.04 (.03)	-1.36	.173		
Age	-.07 (02)	-2.77	.006		
Gender	-.03 (02)	-.89	.372		
Education	-.16 (.02)	-6.04	<.001		

* p<.05, ** p<.01, *** p<.001
SE=standard error, CI=confidence interval, CIT=compulsivity
Models: model-based index ~ CIT + age + gender + education
Gender: Comparison of cisgender woman with reference to cisgender man
Education: negative beta values reflect those with less education associated with greater MBI deficits

Supplementary References

1 Gillan, C. M., Kosinski, M., Whelan, R., Phelps, E. A. & Daw, N. D. Characterizing a psychiatric symptom dimension related to deficits in goal-directed control. *elife* **5**, e11305 (2016). https://doi.org:10.7554/eLife.11305

2 Seow, T. X. F., O'Connell, R. & Gillan, C. M. Model-based learning deficits in compulsivity are linked to faulty representations of task structure. *bioRxiv* (2020). https://doi.org:10.1101/2020.06.11.147447

3 Rouault, M., Seow, T., Gillan, C. M. & Fleming, S. M. Psychiatric Symptom Dimensions Are Associated With Dissociable Shifts in Metacognition but Not Task Performance. *Biological Psychiatry* **84**, 443-451 (2018). https://doi.org:10.1016/j.biopsych.2017.12.017

4 Brown, V. M., Chen, J., Gillan, C. M. & Price, R. B. Improving the Reliability of Computational Analyses: Model-Based Planning and Its Relationship With Compulsivity. *Biological Psychiatry: Cognitive Neuroscience and Neuroimaging* **5**, 601-609 (2020). https://doi.org:10.1016/j.bpsc.2019.12.019

5 Gillan, C. M., Otto, A. R., Phelps, E. A. & Daw, N. D. Model-based learning protects against forming habits. *Cognitive, Affective, & Behavioral Neuroscience* **15**, 523-536 (2015). https://doi.org:10.3758/s13415-015-0347-6

6 Daw, N. D., Gershman, S. J., Seymour, B., Dayan, P. & Dolan, R. J. Model-based influences on humans' choices and striatal prediction errors. *Neuron* **69**, 1204-1215 (2011). https://doi.org:10.1016/j.neuron.2011.02.027

7 Otto, A. R., Gershman, S. J., Markman, A. B. & Daw, N. D. The curse of planning: dissecting multiple reinforcement-learning systems by taxing the central executive. *Psychological science* **24**, 751-761 (2013). https://doi.org:10.1177/0956797612463080

8 Decker, J. H., Otto, A. R., Daw, N. D. & Hartley, C. A. From Creatures of Habit to Goal-Directed Learners: Tracking the Developmental Emergence of Model-Based Reinforcement Learning. *Psychological science* **27**, 848-858 (2016). https://doi.org:10.1177/0956797616639301

9 Haines, N., Kvam, P. D., Irving, L., Tuker-Smith, C., Beauchaine, T. P., Pitt, M. A., Ahn, WY., Turner, B. M. Theoretically Informed Generative Models Can Advance the Psychological and Brain Sciences: Lessons from the Reliability Paradox. *PrePrint* (2020).
