# Supplementary material for: Using smartphones to optimise and scale-up the assessment of model-based planning
Source: Commun Psychol. 2023 Nov 1;1:31. doi: 10.1038/s44271-023-00031-y (PMC11332031; doi:10.1038/s44271-023-00031-y)
Supplement: Supplementary file 3 — Reporting Summary [file 44271_2023_31_MOESM3_ESM.pdf]

nature portfolio

Corresponding author(s): Claire Gillan

Last updated by author(s): Oct 2, 2023

Reporting Summary

Nature Portfolio wishes to improve the reproducibility of the work that we publish. This form provides structure for consistency and transparency in reporting. For further information on Nature Portfolio policies, see our Editorial Policies and the Editorial Policy Checklist.

Statistics

For all statistical analyses, confirm that the following items are present in the figure legend, table legend, main text, or Methods section.

n/a	Confirmed	
☐	☒	The exact sample size (*n*) for each experimental group/condition, given as a discrete number and unit of measurement
☐	☒	A statement on whether measurements were taken from distinct samples or whether the same sample was measured repeatedly
☐	☒	The statistical test(s) used AND whether they are one- or two-sided<br>*Only common tests should be described solely by name; describe more complex techniques in the Methods section.*
☐	☒	A description of all covariates tested
☐	☒	A description of any assumptions or corrections, such as tests of normality and adjustment for multiple comparisons
☐	☒	A full description of the statistical parameters including central tendency (e.g. means) or other basic estimates (e.g. regression coefficient) AND variation (e.g. standard deviation) or associated estimates of uncertainty (e.g. confidence intervals)
☐	☒	For null hypothesis testing, the test statistic (e.g. *F*, *t*, *r*) with confidence intervals, effect sizes, degrees of freedom and *P* value noted<br>*Give P values as exact values whenever suitable.*
☐	☒	For Bayesian analysis, information on the choice of priors and Markov chain Monte Carlo settings
☐	☒	For hierarchical and complex designs, identification of the appropriate level for tests and full reporting of outcomes
☐	☒	Estimates of effect sizes (e.g. Cohen's *d*, Pearson's *r*), indicating how they were calculated

Our web collection on statistics for biologists contains articles on many of the points above.

Software and code

Policy information about availability of computer code

Data collection	Data was collected fully online from the non-profit smartphone application Neureka that was developed and is maintained by the Gillan Lab, Trinity College Dublin. Neureka is freely available to download on Android and iOS app stores.
Data analysis	Data and code to reproduce the main findings and figures are available at: https://osf.io/arhng/. All analysis were performed through RStudio version 1.4.1106 (http://cran.us.r-project.org)

For manuscripts utilizing custom algorithms or software that are central to the research but not yet described in published literature, software must be made available to editors and reviewers. We strongly encourage code deposition in a community repository (e.g. GitHub). See the Nature Portfolio guidelines for submitting code & software for further information.

Data

Policy information about availability of data

All manuscripts must include a data availability statement. This statement should provide the following information, where applicable:

- Accession codes, unique identifiers, or web links for publicly available datasets
- A description of any restrictions on data availability
- For clinical datasets or third party data, please ensure that the statement adheres to our policy

The processed data that supports the findings of this study are available at https://osf.io/arhng/. Due to the sensitive nature of this data (i.e., responses to mental health questionnaire items) and to comply with data protection regulation, there are restrictions to the availability of the unprocessed task and mental health data.

The unprocessed data however can be made available from the authors upon request through the corresponding author. The data sourced from publicly available data sets can be found at https://osf.io/usdgt/3 and https://osf.io/mx9kf/7.

Human research participants

Policy information about studies involving human research participants and Sex and Gender in Research.

Reporting on sex and gender	We only report on and test for gender in this research. Gender-identification was collected in-app by asking 'What gender do you most identify with" and a list of seven options: male, female, transgender male, transgender female, non-binary, not-listed, or prefer not to say. We used gender in a regression analysis to assess associations with individual differences of model-based planning. We also used gender, along with age and education, to control for as covariates in regression models assessing the association between model-based planning and self-report clinical differences.
Population characteristics	Sample characteristics for the sample collected for Experiment 1 included 57 individuals, aged between 18-46 with a mean age of 22.95 years (±5.6 years). The sample included 43 women (66%) and 14 men (34%).<br><br>Sample characteristics for the sample collected for Experiment 2 included 5005 individuals, ranging from 18-82 with a mean age of 45.69 years (±15.54 years). The sample included 3226 (64%) identifying as cis-gender women, 1683 (34%) identifying as cisgender men, 82 (1.9%) identifying as non-cisgender, and 15 (>.01%) who preferred not to disclose their gender identification.<br><br>Information on race and ethnicity was not collected.
Recruitment	Participants were recruited for Experiment 1 through information flyers, research participation pools, and word of mouth at Trinity College Dublin. Participants were compensated with a €10 euro online voucher for their participation.<br>Participants were recruited for Experiment 2 through global citizen science initiatives and digital advertisements for example GoogleAds. Recruitment was completed fully online.
Ethics oversight	Both experiments were granted ethical approval by the School of Psychology Ethics Committee, Trinity College Dublin (Ethical Approval Code: SPREC072019-01). All participants gave online informed consent through the smatphone app prior to participating in this research.

Note that full information on the approval of the study protocol must also be provided in the manuscript.

Field-specific reporting

Please select the one below that is the best fit for your research. If you are not sure, read the appropriate sections before making your selection.

☐ Life sciences ☒ Behavioural & social sciences ☐ Ecological, evolutionary & environmental sciences

For a reference copy of the document with all sections, see nature.com/documents/nr-reporting-summary-flat.pdf

Behavioural & social sciences study design

All studies must disclose on these points even when the disclosure is negative.

Study description	Experiment 1 was a cross-sectional quantitative experimental study. Experiment 2 was quantitative experimental study which included cross-sectional but also longitudinal analyses including test re-test reliability estimates.
Research sample	Experimental 1 recruited a university-based sample from Trinity College Dublin for convenience reasoning. This included 57 participants, aged between 18-46 with a mean of 22.95 years (±5.6 years). Of the 57 participants, 43 identified female (66%) and 14 identified as males (34%). Experiment 2 recruited a population-based sample from all over the world in order to have enough variance to complete individual-difference testing for example a broader age range. We collected data from 5005 participants, aged between 18-82 with a mean of 45.69 (±15.54 years). This sample included 3226 (64%) identifying as cisgender women, 1683 (34%) identifying as cisgender men, 82 (1.9%) identifying as non-cisgender, and 15 (>.01%) who preferred not to disclose their gender identification.
Sampling strategy	In Experiment 1, a priori power analysis revealed a minimum sample size of N=50 was needed to detect a medium effect with 80% power and significance level set at p<.05. We aimed to recruit additional data to account for data loss and exclusions. In Experiment 2, a prior power analysis (based on a prior paper) indicated that a sample of N=541 participants was required to to detect a small effect with 80% power using an online sample.
Data collection	For Experiment 1, data was collected online. During the sign-up process, they provided electronic consent, along with self-reporting basic demographic (age, gender, education) and eligibility information. They completed the traditional two-step task in a web-browser on a laptop or desktop computer and Cannon Blast through the Neureka app on an iOS or android smartphone. For Experiment 2, data was also collected fully online. Here we collected data from general citizen scientist users of the Neureka app. At registration, users provided electronic consent, along with self-reporting socio-demographic information. They then completed Cannon Blast on their smartphone device.

Timing	For both experiments, data was collected between June 2020 (when the Neureka app launched on iOS and android app store platforms) and October 2022.
Data exclusions	For Experiment 1, participants were excluded from the traditional task if they: (a) missed more than 20% of trials (N=2), (b) responded with the same key press at the first stage of the task on more than 95% of trials (N=5). This was based on pre-established criteria. Exclusion criteria for Cannon Blast were harmonised with these as much as possible. We excluded participants if they had (a) missed more than 20% of trials (N=1) or (b) selected the same container more than 95% of the time (N=4). We noted that some trials were missing for 2 users from our app database (presumably due to a technical glitch) and for one of these, this exceeded the 20% threshold and were therefore excluded. Combining all exclusion criteria for both tasks, N=11 (16%) participants were excluded with N=57 remaining for analysis. For Experiment 2, participants were excluded for (a) missing more than 20% trials on their first session (N=2394, most of whom started, but did not complete the game), and (b) selecting the same container on more than 95% of the trials (N=797). A further N=48 were excluded for having incomplete demographic data required for external validation leaving N=5005 remaining for analysis (33% data loss).
Non-participation	In Experiment 1, no participants dropped out prematurely or requested to withdraw. In Experiment 2, 2394 participants were excluded due to insufficient data which could be explained by a) dropping out prematurely or b) technical glitch. As we collect data online and no communication with the participant, we can not estimate a precise number for non-participation in Experiment 2.
Randomization	In experiment 1, all participants completed both task versions the traditional two step task completed on a computer and the gamified task completed on a smartphone. Participant were randomly assigned to complete the computer version of the smartphone version first to reduce any impact of order on the results. In experiment 2, we conducted a series of between subject task-manipulations (transition ratio and reward drift set). For transition ratio, the initial set of participants (N=2884) experienced the 80:20 transition ratio. In order to test the effect of transition ratio, we decided to make a change in-app to set the transition ratio to 70:30 (as in the original computerised task version) and continue to collect data. As fewer participants experienced the 70:30 (N=2138) transition compared to the 80:20 (N=2884), we down-sampled the 80:20 group and propensity score matched them for age, gender and education in order to retrieve two equal sized matched groups (N=2138 in both groups). For reward drifts, participants were randomly assigned to one of two conditions (Drift A or Drift B) in each block of trials.

Reporting for specific materials, systems and methods

We require information from authors about some types of materials, experimental systems and methods used in many studies. Here, indicate whether each material, system or method listed is relevant to your study. If you are not sure if a list item applies to your research, read the appropriate section before selecting a response.

Materials & experimental systems

n/a	Involved in the study
☒	☐ Antibodies
☒	☐ Eukaryotic cell lines
☒	☐ Palaeontology and archaeology
☒	☐ Animals and other organisms
☒	☐ Clinical data
☒	☐ Dual use research of concern

Methods

n/a	Involved in the study
☒	☐ ChIP-seq
☒	☐ Flow cytometry
☒	☐ MRI-based neuroimaging
